# Nature-based and geo-engineering climate mitigation technologies: Public acceptance and security prospects

**DOI:** 10.1016/j.isci.2025.112303

**Published:** 2025-03-27

**Authors:** Nik Hynek, Beata Gavurova, Vaclav Moravec, Matus Kubak

**Affiliations:** 1 Department of Security Studies, Faculty of Social Sciences, Charles University in Prague, Prague, Czechia; 2 Technical University of Košice, Faculty of Mining, Ecology, Process Control and Geotechnologies, Košice, Slovakia; 3 Department of Journalism, Faculty of Social Sciences Charles University in Prague, Prague, Czechia; 4 Technical University of Košice, Faculty of Economics, Košice, Slovakia

**Keywords:** Environmental science, Environmental policy

## Abstract

Climate change requires mitigation approaches, from nature-based to experimental geoengineering. We examined public attitudes toward six strategies—reforestation in previously forested areas, afforestation in new terrains, direct CO_2_ capture with underground storage, biomass energy with CO_2_ capture, stratospheric sulfate aerosols, and orbital mirrors—via a representative Czech survey (*N* = 3,007). Binary logistic regressions reveal how age, education, employment, and residence shape perceptions of efficacy, risks, and ethics. Results show strong favor for reforestation and afforestation due to ecological benefits and long-term promise; sulfate aerosols and orbital mirrors face skepticism. Surprisingly, participants with only primary education showed greater openness to geoengineering than university graduates. Older respondents favored biomass-based carbon capture but less so certain high-tech solutions. Our findings highlight the importance of policies aligned with diverse public views, ensuring both established and novel measures are harmonized into an effective climate mitigation strategy. These results indicate demographic contexts shape acceptance of climate interventions.

## Highlights

• Public attitudes strongly favor nature-based climate solutions like reforestation
• Geoengineering methods face skepticism due to ethical and environmental concerns
• Sociodemographic factors like education and region shape climate-tech perceptions
• Logistic regression models show public support for varied climate mitigation methods

## Graphical abstract

**Figure:**
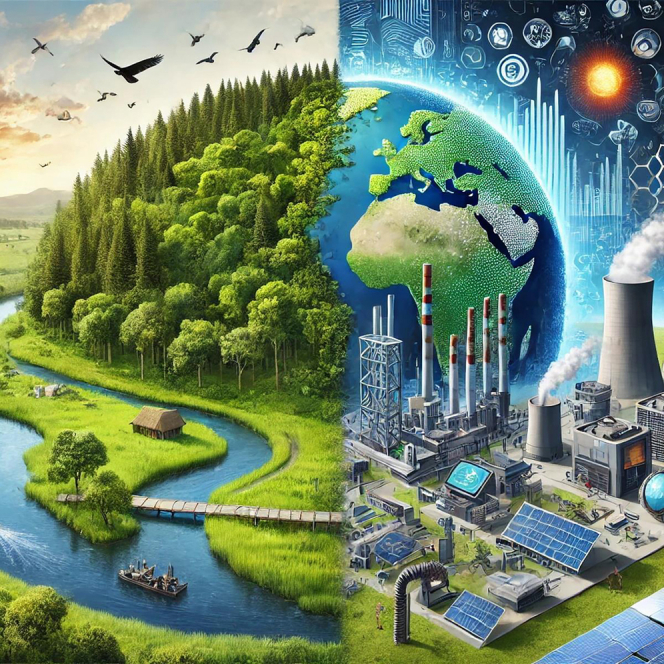

## Introduction

Investigating climate change transcends mere analysis of geophysical variables, increasingly spotlighting the social dynamics it engenders among individuals and communities. This evolution reflects a growing recognition within research circles of climate change’s pervasive impact, from personal experiences to societal structures. The imperative for social sciences to delve into climate change’s multifaceted nature is underscored by emerging critiques of disconnected public discourse and the inadequacy of institutional frameworks in addressing the climate crisis.^1^^,^^2^^,^^3^ The urgency of adapting to rapid climate shifts necessitates a deep understanding of societal attitudes and perceptions toward climate change, advocating for comprehensive strategies that enhance communal resilience against climate variability. Emphasizing the importance of inclusivity, these efforts must account for the diverse coping mechanisms and adaptative capacities across different socio-economic and geographical landscapes.^4^

Driven by the imperative to align public engagement with Sustainable Development Goal 13 (SDG13: Climate Action), this study investigates public attitudes and perceptions toward technologies aimed at mitigating climate change. Recognizing the diversity in perspectives across governance levels, environmental organizations, the business sector, and communities affected by environmental changes, our research addresses the varied ways in which these stakeholders interact with climate change issues. Our analysis offers invaluable insights for policymakers, environmental strategists, and educators by illuminating public sentiments on climate mitigation technologies. The findings are poised to enhance environmental literacy, induce pro-environmental behavior, and inform efforts to combat climate mis/disinformation. Additionally, our work supports the creation of targeted pro-environmental initiatives, contributing to a broader strategy to address climate change and its impacts across different societal segments.

Technological advancements and escalating global climate risks necessitate comprehensive responses across institutional and individual levels to mitigate climate change impacts effectively. Understanding complex ecosystems and societal adaptation to climate change is pivotal for crafting optimal policies, necessitating wide-ranging studies that incorporate geographical, socio-political, and anthropological insights to enhance environmental literacy. Recent decades have underscored the profound effects of climate change on European ecosystems, with significant threats to biodiversity and ecosystem functionality anticipated.^5^ Climate-induced phenomena such as floods, droughts, and forest fires profoundly affect societal functioning, health outcomes, agricultural productivity, and water resources.^6^ Urbanization further exacerbates these challenges, highlighting the urgent need for innovative approaches like nature-based solutions (NbS),^7^ socio-economic path models,^8^ integrated assessment models, or ecosystem adaptation approaches to achieve biodiversity restoration and ecosystem services optimization.^9^ Additionally, understanding the limitations of societal adaptation—rooted in biological, economic, or technological factors—is critical.^10^ These constraints, intertwined with societal values, knowledge, and cultural attitudes toward risk, underscore the necessity for integrated social and technological innovation in climate adaptation strategies.

The discourse on societal adaptation to climate change pivots on strategies to facilitate adjustment as generations face a progressively altering environment.^11^ Critiques of mitigation efforts underscore the importance of recognizing adaptation’s ecological, economic, technological, and physical constraints, which Adger et al.^10^ emphasize for effective policy evaluation. Research by Ackerman et al.,^12^ Eriksen et al.,^13^ and Serrao-Neumann et al.^14^ explore these limitations, while Gallopin^15^ and Nelson et al.^16^ link adaptation to changes in social systems and intentions. O’Brien^17^ further investigates the variability of social values in this context. Contrary to viewing adaptation as merely natural or reactive, Adger et al.^10^ argue for a deeper understanding of societal organization, values, and knowledge exchange. They posit that adaptation limits are inherently societal, influenced by internal factors like ethics, culture, and risk perception, advocating for an exploration of adaptation as a socially constructed process.^18^

The research landscape reveals distinct perspectives on climate adaptation, contrasting risk tolerance approaches with those advocating for societal well-being enhancement.^19^ Stakeholder values diverge based on their power and influence. Adaptation faces constraints from uncertainties in climate change predictions, deriving from limited scientific insights or the ambiguous nature of climate models.^20^ Individual and social factors—risk perception, social standing, age, and personal habits—further influence adaptation responses, potentially hindering collective decision-making processes at the policy level, where inertia and cultural factors play significant roles. Individual perceptions and societal factors shape future climate change decision-making, underscoring the importance of recognizing cognitive and epistemological differences in adaptive strategies.^21^ While current discourse often prioritizes biophysical and economic analyses, it overlooks the geographical, social, and cultural localization of communities affected by climate change.^22^ Stern^23^ argues for considering nonmarket factors in adaptation studies, noting the significance of conceptual, practical, and ethical boundaries. Turner et al.^24^ emphasize the role of externalities and “invisible losses” in adaptation, beyond mere economic considerations. Adger et al.^10^ question assumptions that adaptation is overly constrained by external limitations, suggesting that societal values, perceptions, and power structures are more definitive barriers. This variability across societies indicates that what limits one may not limit another, pointing to the need for systematic exploration of adaptation process assumptions.

This backdrop sets the stage for our research, which fills a gap by examining public attitudes toward climate change mitigation technologies. Despite the heterogeneity of existing studies, our work aims to uncover fundamental conceptual structures, contributing to the development of a comprehensive environmental literacy system.

### Data and methodology

The study, conceptualized by CEDMO (Central European Digital Media Observatory) Charles University and leveraging data from the CEDMO NPO (National Recovery Plan) project, utilized the polling agency MEDIAN for data collection. Employing the CAWI (computer-assisted web interviewing) technique, the survey targeted a demographically representative sample from MEDIAN’s online panel (quota selection). The research sample was stratified by gender, age (16+ years), education, economic status/activity, internet use, region, and size of the place of residence. The research sample consisted of 3,007 respondents, with a slight female majority (1,537; 51.1%) compared to males (1,470; 48.9%). Regarding age distribution, the largest demographic group comprised respondents aged 65 and older (24.6%), followed by those aged 35–44 (18.1%) and 45–54 (17.9%). The age groups 25–34 (14.8%) and 55–64 (14.7%) showed similar representation, while the smallest proportion of respondents was found in the youngest category, aged 16–24 (9.9%). Educational background varied, with the majority having completed high school: 34.9% held a high school diploma and 33.3% had completed high school without graduation. Additionally, 18.9% possessed a university degree and 12.9% had completed only primary education. Economic activity among respondents showed that 46.0% were employed, 12.4% identified as entrepreneurs, and 3.2% were unemployed. Retired individuals constituted 26.5% of the sample, students made up 7.7%, and 4.2% were classified under other economic statuses. Concerning residence size, 39.2% of respondents lived in towns with fewer than 5,000 inhabitants, 38.1% resided in medium-sized towns (5,000–99,999 inhabitants), and 22.7% lived in cities with populations exceeding 100,000. All analyses were conducted using IBM SPSS (International Business Machines Statistical Product and Service Solutions) Statistics, version 30.^25^

Ensuring participant anonymity and the option for withdrawal at any point, the research aimed to mirror the Czech Republic’s demographic makeup closely. This research probes the perceptions of Czech citizens regarding the adoption of climate change mitigation technologies. It assesses public sentiment toward integrating these technologies into efforts to mitigate the adverse effects of climate change. Respondents evaluated a range of statements on technologies using a semantic differential scale, ranging from −6 for complete disagreement to +6 for full agreement.

Our study focused on six distinct climate change mitigation technologies, categorized into three groups for analysis: firstly, two NbS (1 + 2); secondly, two CO_2_-storage technologies, each integrated into current industrial processes (3 + 4); and thirdly, two speculative technologies not yet implemented (5 + 6). This classification aimed to encompass a broad spectrum of approaches, from the natural to the industrial and the innovative, to understand public perception across different technology readiness levels. Opinion poll questions related to each technology were accompanied by detailed visual graphics illustrating their operation to facilitate respondents’ understanding.

For each climate change mitigation technology discussed, we provided participants with comprehensive descriptions alongside visual memos. These visuals were carefully designed to capture the essence of each technology, complementing the textual information and significantly enhancing comprehension among respondents.

The descriptions of the six technologies are contained as follows.

(1) Reforestation or thickening in originally forested areas—biological sequestration of atmospheric carbon; slowing or reversing the release of carbon stored in exposed forest soil into the atmosphere (bark beetle, fire, and excessive logging); and balancing (carbon offsetting) industrial production of CO_2_
(2) Afforestation of new, i.e., originally unforested, areas—biological sequestration of atmospheric carbon and thus balancing (carbon offsetting) industrial CO_2_ production.
(3) Direct capture of CO_2_ from the atmosphere and its underground storage—a technology that embeds CO_2_ in subterranean geological and hydrological layers and can be physically linked to the exploitation of oil deposits.
(4) Energy production from biomass with CO_2_ capture and subsequent underground storage—the grown biomass removes CO_2_ from the air and its subsequent transport and incineration produces renewable energy. Instead of being released into the atmosphere, the CO_2_ is captured and stored indefinitely in underground reservoirs.
(5) Stratospheric sulfate aerosol dispersal—this technology involves the man-made dispersal of small reflective particles into the stratosphere using balloons; it is sometimes referred to as part of the so-called global dimming (similar effect to the so-called volcanic winter), which reduces the amount of radiation falling on the Earth’s surface.
(6) Reflective mirrors in outer space—this technology involves placing reflective structures (mirrors) in orbit around the Earth. The aim is to cool the planet by reflecting incoming solar radiation back into space. Spacecraft would be used for transport.

For each of the six climate mitigation technologies, respondents were tasked with evaluating nine distinct areas using a semantic differential scale. These evaluations were directly linked to the previously detailed statements, ensuring a comprehensive assessment framework. Respondents allocated points across this spectrum to express their perceptions and attitudes toward each technology’s effectiveness, ethical implications, feasibility, and other critical aspects outlined in the study’s framework. The domains were as follows.

(1) Well-spent money.
(2) Long-term solution.
(3) Environmentally friendly.
(4) Understandable intention.
(5) Cooperation among states.
(6) Aesthetically hideous.
(7) Disinformation potential.
(8) Dangerous technology.
(9) Interstate conflict escalation.

When developing our research instrument (questionnaire), we drew partial inspiration from the study by Baum et al.,^26^ which uniquely examined public perceptions of climate intervention technologies across thirty countries and nineteen languages. Our research, however, significantly extends their seminal work. Specifically, we conducted our survey in Czechia—a Central European post-communist country not included in the Baum et al.^26^ study—with a sample size approximately three times larger per country, allowing for a more detailed and granular dataset. Furthermore, we introduced four additional analytical dimensions: diplomatic cooperation, implications for international security (including potential escalation of inter-state conflicts), the risk of disinformation adversely impacting information security and political stability, and the clarity of the intended purpose of each technology. Lastly, unlike Baum et al.^25^^,^^26^, our research distinguishes explicitly between afforestation and reforestation, revealing subtle differences in public attitudes toward these two climate mitigation approaches. For the construction of our questions, we have further consulted studies by Pita et al.,^27^ Psistaki et al.^28^ Flight and Tait,^29^ Keith et al.,^30^ Fuss et al.,^31^ Crutzen et al.,^32^ and Baum et al.^33^

## Results

### Opinions on climate mitigation technologies

Table 1 summarizes the findings, presenting average responses from participants on the semantic differential scale, which spans from −6 (indicating full disagreement) to +6 (indicating full agreement).

**Table 1:** Opinions on climate mitigation technologies

	Money well spent	A long-term solution	Favorable for the environment	An understandable intention	Several states can cooperate on this	It’s disgusting	An opportunity for disinformation	Dangerous technology	It can create conflicts between states
Reforestation or thickening of afforestation in originally forested areas (reforestation)	3.49	3.32	3.82	3.40	3.60	−3.91	−1.92	−1.92	−3.21
Afforestation of new, originally unforested areas (afforestation)	2.76	2.82	3.12	2.85	2.88	−3.48	−1.83	−1.83	−2.93
Direct capture of CO2 from the atmosphere and its underground storage	−0.23	0.06	−0.15	−0.28	0.92	−0.82	0.26	0.26	−0.01
Generation of energy from biomass with capture and subsequent underground installation of CO2	0.91	0.96	1.06	0.93	1.43	−1.55	−0.26	−0.26	−0.86
Dispersion of sulfate aerosols in the stratosphere	−1.64	−1.37	−1.48	−1.19	−0.05	−0.31	0.73	0.73	0.41
Deployment of reflective structures (mirrors) on the Earth’s orbit	−2.03	−1.11	−1.34	−1.19	0.64	−0.22	0.97	0.97	0.44

Reforestation, as a climate change mitigation strategy, received highly positive evaluations, with respondents noting its environmental benefits (+3.82), potential for international cooperation (+3.60), viability as a long-term solution (+3.32), and value as a wise investment (+3.49). In contrast, respondents showed minimal concerns regarding disinformation (−1.92) and rarely viewed reforestation as objectionable (−3.91) or likely to cause conflicts (−3.21). Direct CO_2_ capture elicited more neutral reactions, with average ratings reflecting ambivalence toward its environmental benefit (−0.15), moderate optimism about its potential for international cooperation (+0.92), and skepticism regarding cost-effectiveness (−0.23). Biomass energy generation combined with CO_2_ capture and storage was somewhat positively regarded, especially concerning clarity of purpose (+0.93), environmental potential (+1.06), and being money well spent (+0.91), while eliciting relatively low levels of disapproval regarding its general appeal (−1.55). Sulfate aerosol dispersion in the stratosphere received mildly negative evaluations, primarily due to concerns about cost-effectiveness (−1.64), long-term viability (−1.37), and overall environmental benefit (−1.48). Respondents also expressed slight concerns regarding disinformation risks (+0.73). Reflective space mirrors attracted skepticism, particularly related to concerns over misinformation (+0.97) and doubts about economic feasibility (−2.03).

**Figure 1:**
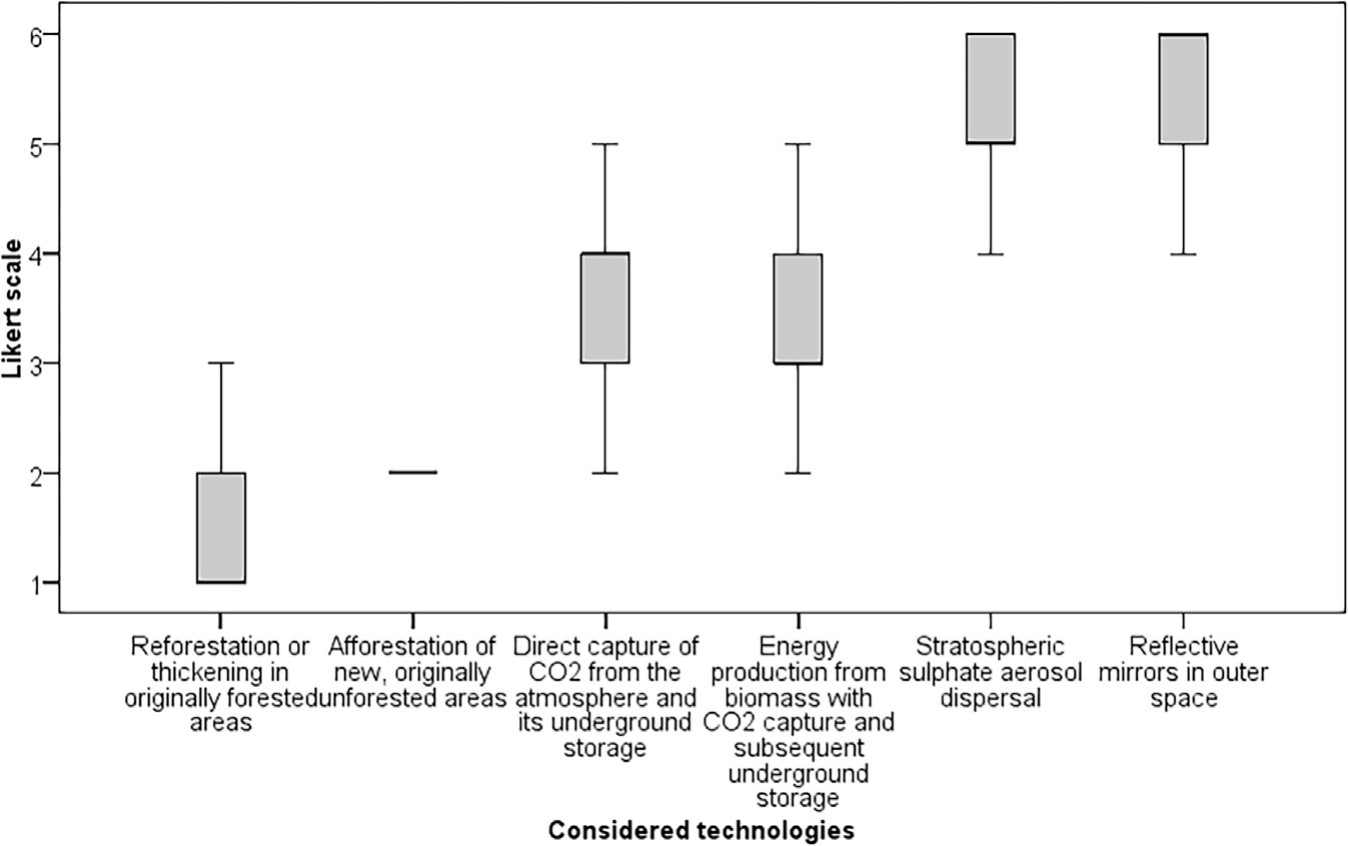
Meaningfulness of climate mitigation technologies

Participants were also asked to evaluate the significance of these technologies on a scale ranging from 1 (most meaningful) to 6 (least meaningful). These evaluations are visually summarized in Figure 1 using boxplots, providing comprehensive insights into respondents’ views. Overall, the survey illustrates a gradient of public sentiment toward climate change mitigation technologies, ranging from broad acceptance of NbS to cautious skepticism toward certain geoengineering approaches.

Reforestation is highly valued, with its efficacy in carbon sequestration and ecosystem restoration reflected in a mean significance score of 2 and a median of 1, highlighting broad agreement on its utility, particularly in originally forested areas. Afforestation, similarly, recognized for introducing forest cover in originally non-forested areas, shares reforestation’s high regard among participants, underscored by a mean and median significance score of 2 and minimal variance, indicating uniform perception. Conversely, direct CO_2_ capture with underground storage is perceived as less critical, evidenced by a lower mean significance score of 4, suggesting it’s seen as a lesser priority relative to natural solutions. Biomass energy generation with carbon capture occupies a middle ground in public perception, with a mean significance score of 3, denoting moderate importance for carbon capture efforts and mirrored variance with direct CO_2_ capture, indicating similar respondent views. Sulfate aerosol dispersion and the deployment of orbital mirrors are deemed least significant, with mean scores of 5 and a slightly higher variance for mirrors indicating a broader dispersion of views. These findings suggest a cautious or skeptical stance toward these technologies, with a clear preference for more traditional, nature-based approaches among the public.

Our analysis continued with six binary logistic regressions to assess how sociodemographic factors shape the perceived meaningfulness of technologies for climate change mitigation. The aim was to predict the odds of a technology being deemed meaningful by respondents. We dichotomized the outcome variable based on the scale ratings for technology significance: Ratings from 1 to 3 were interpreted as the technology being meaningful (assigned a value of 1), whereas ratings from 4 to 6 indicated it as not meaningful (assigned a value of 0). This binary classification facilitated modeling the influence of sociodemographic variables on technology perceptions within the climate change context.

Table 2 illustrates the distribution of opinions regarding the meaningfulness of various climate change mitigation technologies. A preliminary examination of the table reveals a stark disparity in public perception. A substantial number of respondents, totaling 2,803, perceive reforestation as a meaningful climate action, underscoring its recognized importance in carbon sequestration and biodiversity preservation. Similarly, afforestation is deemed meaningful by 2,646 respondents, reflecting its appreciated potential for enhancing forest cover. In contrast, direct CO_2_ capture and underground storage elicit less enthusiasm, with only 881 respondents finding it meaningful, indicating lower public confidence in this technology’s impact on climate mitigation. Meanwhile, biomass energy generation combined with carbon capture and storage is considered meaningful by 2,044 individuals, suggesting a moderate acknowledgment of its potential benefits. Notably, more unconventional approaches like sulfate aerosol dispersion and orbital mirrors attract minimal support, with merely 306 and 342 respondents, respectively, acknowledging their meaningfulness. This indicates a general reluctance to endorse less traditional methods of climate intervention.

**Table 2:** Distribution of dichotomous variable

Considered technology	Meaningfulness	Count
Reforestation or thickening in originally forested areas	not meaningful	204
	meaningful	2,803
Afforestation of new, originally unforested areas	not meaningful	361
	meaningful	2,646
Direct capture of CO_2_ from the atmosphere and its underground storage	not meaningful	2,126
	meaningful	881
Energy production from biomass with CO_2_ capture and subsequent underground storage	not meaningful	963
	meaningful	2,044
Stratospheric sulfate aerosol dispersal	not meaningful	2,701
	meaningful	306
Reflective mirrors in outer space	not meaningful	2,665
	meaningful	342

Binary logistic regression is utilized for analyzing outcomes when the dependent variable is dichotomous.^34^ This approach is particularly suited for scenarios where traditional linear regression’s assumptions do not hold, offering a flexible alternative. The model operates on the principle that the log-odds of the dependent variable can be predicted from a linear combination of independent variables, allowing for the estimation of how various factors influence the likelihood of a particular outcome.

(Equation 1) $$\ln\left(\frac{\mathrm{Pr}\left(Technology\;is\;meaningful\right)}{1-\mathrm{Pr}\left(Technology\;is\;meaningful\right)}\right)=\beta_{0}+\sum_{i=1}^{n}\beta_{i}\times x_{i}$$

where *Pr*(*Technology is meaningful*) represents the likelihood that the respondent considers the technology to be meaningful, while 1−Pr(Technologyismeaningful) indicates the likelihood of the opposite scenario. In this context, β_0_ remains a constant within the model, β_i_ represents the estimated regression coefficients, and x_i_ denotes the set of explanatory variables.

In our study, we conducted six distinct regression models, each focusing on a different climate mitigation technology as identified in Table 2. The independent variables considered in these models include the respondents’ gender, age, education level, living region, residential area size, economic activity, and frequency of internet usage. The regression analyses aim to clarify how these sociodemographic factors influence the perceived meaningfulness of each technology. The results, detailed in subsequent tables, showcase only those variables that proved to be statistically significant. We interpret the significance of the regression model coefficients at the 0.1 significance level. The omnibus test for all regression models presented in the following yields a *p* value of 0.000, indicating that the logistic regression models incorporating the specified predictors are statistically significant. Thus, the inclusion of these predictors provides a significantly better explanation of the dependent variable compared to an intercept-only model.

### Reforestation or thickening in originally forested areas

Table 3 presents the results of a regression predicting the likelihood that respondents consider reforestation or thickening of originally forested areas to be useful, based on their level of education and economic activity.

**Table 3:** Binary logistic regression: Reforestation or thickening in originally forested areas

Parameter	B	SE	Hypothesis test				95% CI for Exp(B)	
			Wald chi-square	df	Sig.	Exp(B)	Lower	Upper
(Intercept)	1.869	0.3938	22.531	1	0.000	6.484	2.997	14.030

**Education**								

Primary school	−0.836	0.2898	8.330	1	0.004	0.433	0.246	0.765
High school without diploma	−0.682	0.2400	8.073	1	0.004	0.506	0.316	0.809
High school with diploma	−0.145	0.2573	0.317	1	0.573	0.865	0.523	1.433
University degree	0^a^	.	.	.	.	1	.	.

**Economic activity**								

Employed	−0.734	0.3558	4.253	1	0.039	0.480	0.239	0.964
Entrepreneur	0.207	0.4694	0.194	1	0.659	1.230	0.490	3.086
Unemployed	−0.495	0.5481	0.817	1	0.366	0.609	0.208	1.784
Retired	−0.787	0.3874	4.128	1	0.042	0.455	0.213	0.973
Student	0^a^	.	.	.	.	1	.	.

Dependent variable: reforestation or thickening in originally forested areas.

Model: (intercept), education, economic activity.

a Set to zero because this parameter is redundant.

The regression model’s intercept is statistically significant, suggesting it plays a meaningful role in predicting the likelihood of reforestation being perceived as valuable for climate change mitigation. This intercept sets the baseline odds of valuing reforestation when other variables are held constant at zero. Specifically, the Exp(B) value of 6.484 implies the baseline odds are approximately 6.484 times greater under these conditions, although interpreting log odds directly for the intercept can be complex. Education level significantly influences perceptions of reforestation’s meaningfulness. Respondents with only primary education are 56.7% less likely to see reforestation as meaningful compared to those with university-level education. Similarly, individuals with high school without diploma have 49.4% lower odds of valuing reforestation compared to university graduates, highlighting a clear educational gradient in environmental values. However, the difference for high school graduates does not reach statistical significance. In terms of economic activity, employed individuals and retirees exhibit lower probabilities of considering reforestation meaningful—52% and 54.5% less likely, respectively, compared to students.

### Afforestation of new, originally unforested areas

Table 4 draw the significant factors influencing the perception of afforestation in previously unforested areas as a meaningful climate change mitigation strategy. The statistical significance of the intercept (*p* value < 0.000) underscores a baseline inclination among respondents to view afforestation favorably when other variables are held at zero. Gender plays a notable role, with males demonstrating a 20% lower likelihood than females to perceive afforestation as meaningful. Education level further differentiates attitudes: respondents completing high schools without diploma are 26.4% less likely to value afforestation compared to those with a university degree. Geographic location also influences perceptions; individuals in “rest of the Czech Republic” category are 30.9% less inclined to see afforestation as meaningful compared to Moravian residents. Interestingly, residents of mid-sized cities (population 5,000 to 99,999) are 75.3% more likely to consider afforestation meaningful than those from larger cities (population 100,000+). Entrepreneurial respondents exhibit a significantly higher appreciation for afforestation, being 3.6 times more likely to find it meaningful than students.

**Table 4:** Binary logistic regression: Afforestation of new, originally unforested areas

Parameter	B	SE	Hypothesis test				95% CI for Exp(B)	
			Wald chi-square	df	Sig.	Exp(B)	Lower	Upper
(Intercept)	1.886	0.4258	19.624	1	0.000	6.595	2.863	15.195

**Gender**								

Male	−0.214	0.1195	3.197	1	0.074	0.808	0.639	1.021
Female	0^a^	.	.	.	.	1	.	.

**Education**								

Primary school	−0.328	0.2206	2.213	1	0.137	0.720	0.467	1.110
High school without diploma	−0.306	0.1766	3.008	1	0.083	0.736	0.521	1.041
High school with diploma	0.052	0.1842	0.081	1	0.777	1.054	0.734	1.512
University degree	0^a^	.	.	.	.	1	.	.

**Region**								

Prague and Central Bohemia	−0.007	0.1613	0.002	1	0.966	0.993	0.724	1.362
Rest of the Czech Republic	−0.370	0.1380	7.186	1	0.007	0.691	0.527	0.905
Moravia	0^a^	.	.	.	.	1	.	.

**Size of the place of residence**								

<4 999	0.070	0.1580	0.194	1	0.659	1.072	0.787	1.461
5 000–99 999	0.561	0.1744	10.357	1	0.001	1.753	1.245	2.467
100 000+	0^a^	.	.	.	.	1	.	.

**Economic activity**								

Employed	0.226	0.2243	1.013	1	0.314	1.253	0.807	1.945
Entrepreneur	1.279	0.3276	15.247	1	0.000	3.594	1.891	6.831
Unemployed	0.519	0.4005	1.676	1	0.195	1.680	0.766	3.682
Retired	0.130	0.2351	0.306	1	0.580	1.139	0.718	1.806
Student	0^a^	.	.	.	.	1	.	.

Dependent variable: afforestation of new, originally unforested areas.

Model: (intercept), gender, education, region, size of the place of residence, economic activity.

a Set to zero because this parameter is redundant.

### Direct capture of CO_2_ from the atmosphere and its underground storage

Table 5 delineates the sociodemographic determinants impacting perceptions of direct CO_2_ capture from the atmosphere and its underground storage as a viable climate change solution. The intercept’s statistical significance underscores a baseline inclination toward viewing this technology as meaningful in the absence of other predictors. Age emerges as a subtle yet significant factor, with a slight decrease in the odds (approximately 0.9%) of valuing this technology for every year increase in age, suggesting younger individuals might be more receptive to such innovative approaches. Interestingly, respondents with primary education are about 1.42 times more likely to regard direct CO_2_ capture and storage as meaningful compared to university-educated respondents. Geographical location also plays a role; individuals from Prague and Central Bohemia are less likely, with an odds ratio of 0.64, to consider this technology meaningful compared to those from Moravia. Conversely, respondents from other areas of the Czech Republic show a higher likelihood (odds ratio of 1.23) of valuing direct CO_2_ capture and storage more than Moravians, reflecting regional differences in environmental priorities or awareness. Urbanization level influences perceptions as well; residents of medium-sized cities (populations between 5,000 and 99,999) are 0.77 times less inclined to see direct CO_2_ capture and storage as meaningful compared to those from larger cities.

**Table 5:** Binary logistic regression: Direct capture of CO_2_ from the atmosphere and its underground storage

Parameter	B	SE	Hypothesis test				95% CI for Exp(B)	
			Wald chi-square	df	Sig.	Exp(B)	Lower	Upper
(Intercept)	−0.512	0.2621	3.820	1	0.051	0.599	0.358	1.001
Age	−0.009	0.0032	7.269	1	0.007	0.991	0.985	0.998

**Education**								

Primary school	0.349	0.1502	5.406	1	0.020	1.418	1.056	1.904
High school without diploma	0.063	0.1276	0.243	1	0.622	1.065	0.829	1.367
High school with diploma	0.198	0.1257	2.490	1	0.115	1.219	0.953	1.560
University degree	0^a^	.	.	.	.	1	.	.

**Region**								

Prague and Central Bohemia	−0.450	0.1177	14.604	1	0.000	0.638	0.506	0.803
The rest of the Czech Republic	0.207	0.0977	4.492	1	0.034	1.230	1.016	1.490
Moravia	0^a^	.	.	.	.	1	.	.

**Size of the place of residence**								

<4 999	−0.098	0.1197	0.670	1	0.413	0.907	0.717	1.146
5 000–99 999	−0.266	0.1257	4.484	1	0.034	0.766	0.599	0.980
100 000+	0^a^	.	.	.	.	1	.	.

Dependent variable: direct capture of CO_2_ from the atmosphere and its underground storage.

Model: (intercept), age, education, region, size of the place of residence.

a Set to zero because this parameter is redundant.

### Energy production from biomass with CO_2_ capture and subsequent underground storage

Table 6 sheds light on the factors influencing views on the meaningfulness of biomass energy production combined with CO_2_ capture and underground storage as a climate change mitigation strategy. Interestingly, an analysis reveals that with each incremental year in age, the likelihood of deeming this technology as impactful slightly increases by 1.2% (odds ratio of 1.012), indicating age-related trend toward its acceptance. Education significantly affects these perceptions. Respondents with primary education demonstrate notably lower odds (odds ratio of 0.576) of recognizing the importance of biomass energy with CO_2_ capture compared to their university-educated counterparts, marking a pronounced disparity in environmental technology perceptions across educational levels. Similarly, those with high school education, regardless of obtaining a diploma, are less inclined (odds ratios of 0.708 and 0.670, respectively) to view this technology as meaningful against when compared to university degree holders.

**Table 6:** Binary logistic regression: Energy production from biomass with CO_2_ capture and subsequent underground storage

Parameter	B	SE	Hypothesis test				95% CI for Exp(B)	
			Wald chi-square	df	Sig.	Exp(B)	Lower	Upper
(Intercept)	0.519	0.1469	12.469	1	0.000	1.680	1.260	2.240
Age	0.012	0.0024	23.282	1	0.000	1.012	1.007	1.017

**Education**								

Primary school	−0.552	0.1466	14.161	1	0.000	0.576	0.432	0.768
High school without diploma	−0.345	0.1234	7.827	1	0.005	0.708	0.556	0.902
High school with diploma	−0.400	0.1226	10.662	1	0.001	0.670	0.527	0.852
University degree	0^a^	.	.	.	.	1	.	.

Dependent variable: energy production from biomass with CO_2_ capture and subsequent underground storage.

Model: (intercept), age, education.

a Set to zero because this parameter is redundant.

### Stratospheric sulfate aerosol dispersal

In Table 7, our binary logistic regression analysis reveals the sociodemographic factors affecting views on the significance of deploying sulfate aerosols in the stratosphere to address climate change. Age negatively impacts the perception of this technology’s meaningfulness, with the analysis showing a 2.2% decrease in the odds of favorability for every additional year (odds ratio of 0.978). Education levels significantly influence attitudes toward this geoengineering approach. Respondents with primary education are 1.73 times more likely to view the use of sulfate aerosols as meaningful (odds ratio of 1.729) in comparison to university graduates. Those with high school education without diploma also show a heightened likelihood (odds ratio of 1.543) of endorsing this technology over university graduates. The role of economic activity reveals the following insights: employed participants are 61.1% more inclined to support sulfate aerosol dispersion as a meaningful climate intervention compared to students. Unemployed respondents show an even stronger belief in its efficacy, being twice as likely to endorse it as students. A notable observation is the impact of internet usage frequency: individuals who access the internet several times a day are 31.5% less likely to believe in the technology’s value in combating climate change, underscoring the influence of online information and digital engagement on environmental perceptions.

**Table 7:** Stratospheric sulfate aerosol dispersal

Parameter	B	SE	Hypothesis test				95% CI for Exp(B)	
			Wald chi-square	df	Sig.	Exp(B)	Lower	Upper
(Intercept)	−0.967	0.5625	2.954	1	0.086	0.380	0.126	1.145
Age	−0.022	0.0068	10.766	1	0.001	0.978	0.965	0.991

**Education**								

Primary school	0.548	0.2403	5.196	1	0.023	1.729	1.080	2.770
High school without diploma	0.434	0.1912	5.144	1	0.023	1.543	1.061	2.244
High school with diploma	0.007	0.1996	0.001	1	0.971	1.007	0.681	1.490
University degree	0^a^	.	.	.	.	1	.	.

**Region**								

Prague and Central Bohemia	0.197	0.1639	1.444	1	0.230	1.218	0.883	1.679
The rest of the Czech Republic	0.257	0.1491	2.975	1	0.085	1.293	0.966	1.733
Moravia	0^a^	.	.	.	.	1	.	.

**Economic activity**								

Employed	0.477	0.2531	3.552	1	0.059	1.611	0.981	2.646
Entrepreneur	−0.396	0.3360	1.392	1	0.238	0.673	0.348	1.300
Unemployed	0.694	0.3754	3.415	1	0.065	2.001	0.959	4.177
Retired	0.138	0.2742	0.255	1	0.614	1.148	0.671	1.966
Student	0^a^	.	.	.	.	1	.	.

**Frequency of internet use**								

Several times a day	−0.379	0.2205	2.949	1	0.086	0.685	0.444	1.055
Once a day or almost daily	−0.077	0.2109	0.134	1	0.714	0.926	0.612	1.399
Less often	0^a^	.	.	.	.	1	.	.

Dependent variable: stratospheric sulfate aerosol dispersal.

Model: (intercept), age education, region, economic activity, frequency of internet use.

a Set to zero because this parameter is redundant.

### Reflective mirrors in outer space

Table 8 presents findings from our regression analysis on how likely respondents are to perceive the deployment of orbital mirrors as an effective method to combat climate change. The analysis highlights notable differences based on gender, education, region, employment status, and urbanization level. Gender emerges as a significant factor, with males being 1.34 times more inclined to view this geoengineering approach as meaningful compared to females. Educational background significantly influences perceptions: individuals with primary education are 4.26 times more likely to endorse this technology’s efficacy than university graduates. Similarly, respondents with high school education obtaining diploma and those who completed high school without diploma are 2.88 and 1.94 times more likely, respectively, to find this method meaningful in addressing climate concerns. Geographical differences reveal that respondents from the category “rest of the Czech Republic” are approximately 0.74 times less likely to consider the technology meaningful compared to those from Moravia region, indicating regional variations in geoengineering attitudes. Employment status also plays a role, with unemployed respondents showing a 3.15 times higher likelihood of support compared to students. Moreover, individuals from smaller cities (population under 100,000) are 0.72 times less likely to consider orbital mirrors a viable solution than those from larger cities, suggesting that urbanization level affects the acceptance of innovative climate change strategies.

**Table 8:** Reflective mirrors in outer space

Parameter	B	SE	Hypothesis test				95% CI for Exp(B)	
			Wald chi-square	df	Sig.	Exp(B)	Lower	Upper
(Intercept)	−1.718	0.3850	19.907	1	0.000	0.179	0.084	0.382

**Gender**								

Male	0.294	0.1245	5.565	1	0.018	1.341	1.051	1.712
Female	0^a^	.	.	.	.	1	.	.

**Education**								

Primary school	1.449	0.2489	33.893	1	0.000	4.259	2.615	6.937
High school without diploma	1.056	0.2130	24.589	1	0.000	2.876	1.894	4.367
High school with diploma	0.660	0.2200	9.013	1	0.003	1.935	1.258	2.979
University degree	0^a^	.	.	.	.	1	.	.

**Region**								

Prague and Central Bohemia	0.187	0.1545	1.460	1	0.227	1.205	0.890	1.632
The rest of the Czech Republic	−0.301	0.1488	4.103	1	0.043	0.740	0.553	0.990
Moravia	0^a^	.	.	.	.	1	.	.

**Economic activity**								

Employed	0.317	0.2386	1.761	1	0.184	1.372	0.860	2.191
Entrepreneur	−0.180	0.3004	0.361	1	0.548	0.835	0.463	1.504
Unemployed	1.148	0.3278	12.261	1	0.000	3.151	1.657	5.990
Retired	0.082	0.2581	0.100	1	0.752	1.085	0.654	1.800
Student	0^a^	.	.	.	.	1	.	.

**Size of the place of residence**								

<4 999	−0.330	0.1652	3.994	1	0.046	0.719	0.520	0.994
5 000–99 999	−0.174	0.1705	1.041	1	0.308	0.840	0.602	1.174
100 000+	0^a^	.	.	.	.	1	.	.

Dependent variable: reflective mirrors in outer space

Model: (intercept), gender, education, region, economic activity, size of the place of residence.

a Set to zero because this parameter is redundant.

## Discussion

Numerous studies on climate change communication have highlighted the segmentation in how different population groups perceive climate risks, indicating varied interpretations of climate frames, goals, messages, and risks.^25^^,^^33^ This diversity in understanding climate change has led to different attitudes toward mitigation tools, a finding corroborated by our study. Our research offers insights into public attitudes toward various climate change mitigation technologies, underscoring the importance of these perceptions in building public support for SDG13-related climate action. NbS like reforestation and afforestation are particularly favored, seen as vital for carbon sequestration and ecosystem restoration. However, skepticism surrounds geoengineering approaches like stratospheric sulfate aerosol dispersion and orbital mirrors, with concerns about their effectiveness and ethical implications suggesting apprehensions about altering natural systems on a large scale. Socio-demographic factors—age, gender, education level, and economic activity—further influence these perceptions. Studies by Capstick et al.^35^ and Wolf and Moser^36^ support the notion that awareness and concerns about climate change vary with education, gender, and other demographic aspects. Notably, Wolf and Moser^27^ found gender differences in scientific knowledge and concern about climate change, with women generally more concerned but underestimating their understanding compared to men. Recognizing these differences is essential for crafting communication strategies and policies that effectively engage all segments of society in climate change mitigation efforts.

Adapting to climate change requires understanding both exogenous influences and crucial social factors impacted by policy. This calls for a detailed examination of diverse population groups’ perceptions, environmental behaviors, and confidence in influencing climate change. The response of individuals, communities, and political entities to climate change also hinges on ideological attitudes.^37^^,^^38^ A resilient society considers climate change impacts in conjunction with social dynamics, emphasizing the needs of vulnerable and underdeveloped regions across all institutional levels. Norris et al.^39^ highlight the critical role of social resilience, defined as the capacity to withstand socio-political and environmental challenges. Looking ahead, it’s crucial to explore the intersection of ethics with adaptation goals and decision-making. Despite the consensus on the necessity for ongoing knowledge exchange about climate impacts, predictability alone will not ease adaptation, which is also shaped by societal values and priorities. Enhancing communication to foster significant social engagement is paramount,^40^ considering both behavioral and political dimensions^41^ to address underlying socio-demographic and political factors. Future initiatives must align human behaviors with information processing regarding climate impact, emphasizing the potential for individual and collective action toward systemic changes. Overcoming widespread skepticism remains a challenge, necessitating strategies that demonstrate effective influence on climate change.^17^^,^^42^

Authors advocate for local adaptation strategies rooted in current scientific knowledge to address climate change, emphasizing the need for strategies that consider stress management and macro-social influences on local institutions.^43^ This approach aims to engage communities actively in risk reduction. Targeted strategies for specific communities and regions can significantly lower their climate vulnerability, a sentiment echoed by Cramer et al.^44^ regarding creating scenarios for at-risk societies. The effectiveness of these strategies hinges on interdisciplinary cooperation, technology scalability, social networks, and behavioral economics,^45^ with digital and IS technologies playing a pivotal role.^46^ Institutional leadership and clear climate policy priorities are vital for enhancing societal engagement in climate action, alongside promoting supportive social norms and identities conducive to environmental stewardship. Such efforts aim to counteract public indifference and ignorance toward climate change.^47^^,^^48^ The media industry’s role in promoting environmental literacy and engagement is also critical, underscoring the need for effective communication strategies.^41^ Additionally, female involvement in climate policy can significantly influence environmental activism and engagement.^49^^,^^50^

The study’s impact on achieving SDG13 includes the following.

(1) Aiding the creation and implementation of measures to combat climate change and its effects.
(2) Enhancing societal resilience and adaptability to climate-related hazards and natural disasters.
(3) Assisting policymakers in integrating climate action into national policies, strategies, and planning.
(4) Initiating improvements in environmental literacy and awareness, and bolstering capacities for climate mitigation, adaptation, and early warning systems.

The study underscores the impact of socio-demographic factors on perceptions and attitudes toward climate change mitigation technologies, alongside varying levels of environmental literacy. It enables the identification of fundamental aspects critical for crafting effective environmental literacy frameworks. Challenges like low environmental literacy, information overload, and significant socio-economic and cultural shifts tend to encourage superficial information processing. This trend hinders the ability to focus on systematic and long-term decision-making, as highlighted by Moser,^41^ emphasizing the importance of addressing these barriers to achieve informed and strategic responses to climate change. The findings stress the dynamic nature of public perceptions on climate change, urging attention to opinion trends and the influence of social forces and misinformation.^33^^,^^42^ Future work will delve deeper into shaping public opinion on climate change, employing qualitative, anthropological, and neuroscience methodologies.^51^^,^^52^

### Conclusion

Our exploration into public attitudes toward climate change mitigation technologies uncovered a multifaceted spectrum of perceptions and apprehensions. Significantly, there was a pronounced endorsement for reforestation and afforestation, recognized widely as viable methods for addressing climate change. These NbS garnered positive reception for their ecological advantages, capacity for fostering global cooperation, and sustainable impact over time. On the other hand, geoengineering methods, including stratospheric dispersion of sulfate aerosols and the deployment of orbital mirrors, faced notable skepticism and ethical concerns. This dichotomy highlights the varied levels of acceptance and trust in different technological approaches to mitigate climate challenges, reflecting a preference for traditional, earth-centric solutions over more intrusive, technologically advanced interventions.

Our analysis across various climate change mitigation technologies has illuminated the significant influence of sociodemographic factors on public perceptions. In the realm of reforestation and afforestation, it’s clear that an individual’s education level and economic activity play crucial roles in shaping their views on the meaningfulness of enhancing forest coverage in originally forested areas. Similarly, when considering afforestation of new, previously unforested areas, factors such as gender, geographical region, the size of the respondent’s city, and whether they are entrepreneurs, have emerged as key determinants in evaluating the significance of such environmental actions. Furthermore, the perspective on direct capture of CO_2_ from the atmosphere and its storage underground is significantly influenced by the respondent’s age, their level of education—particularly those with primary school education, the region they reside in, and the size of their living area. This pattern of influence extends to the perception of generating energy from biomass with CO_2_ capture and subsequent underground installation, where age and educational attainment stand out as influential factors. Notably, the dispersion of sulfate aerosols in the stratosphere as a means to combat climate change is viewed through the lens of age, education level—with a special emphasis on those without a high school diploma—and the frequency of internet usage, underscoring diverse informational influences on public opinion. Lastly, the deployment of reflective structures (mirrors) in Earth’s orbit introduces gender, educational background (particularly noting primary education and high school education without a diploma), regional factors, unemployment status, and the size of the respondent’s residence as critical in molding perceptions of this futuristic climate mitigation technology.

Our investigation into public attitudes toward climate change mitigation highlights the necessity of aligning climate policies and communication strategies with public perceptions and concerns. Addressing skepticism and engaging in meaningful dialog with stakeholders is essential for navigating public opinion complexities and devising effective climate action plans. The array of opinions and attitudes, influenced by ethical, cultural, and risk considerations, form the basis for adaptive responses to climate change. Decision-making in this realm often relies more on societal risk perceptions than on precise knowledge, suggesting that understanding these perceptions is key to identifying adaptive limits. This knowledge uncovers the underlying values and interests that guide adaptation strategies, emphasizing the importance of considering the political and ideological aspects of climate adaptation and enkindling a collaborative environment for all parties involved. The scientific community, as the custodian of climate knowledge, plays a crucial role in communicating climate issues. Building effective communication channels between scientists and the public is critical for ensuring a well-informed and united approach to tackling climate change, underscoring the importance of mutual understanding and engagement across different societal sectors.

### Limitations of the study

Finally, we acknowledge several limitations that merit consideration in our study. First, the varying degrees of environmental literacy among respondents could have affected their understanding of the studied technologies, potentially influencing their responses and introducing a bias toward more intuitive or familiar methods. Second, the study’s geographical focus exclusively on Czechia limits generalizability, given potential differences in environmental attitudes and cultural contexts elsewhere. Third, using a single-wave data collection prevented the examination of opinion trends and the dynamics of public acceptance over time, which longitudinal research could clarify. Fourth, the absence of qualitative methodologies, such as focus groups, limited our capacity to explore deeper motivations behind respondents’ perceptions, potentially overlooking valuable insights into underlying beliefs. Last, our analysis included only six selected technologies and did not cover other existing or emerging methods, nor did it incorporate a broader set of attributes for each technology that could provide more detailed understandings of public acceptance. Additionally, potential social desirability bias inherent in survey responses may have influenced participants to respond in ways perceived as more environmentally responsible.

## Resource availability

### Lead contact

Further information and requests for resources should be directed to and will be fulfilled by the lead contact, Beata Gavurova (beata.gavurova@tuke.sk).

### Materials availability

All models generated in this study are available from the lead contact with a completed materials transfer agreement.

### Data and code availability

• All data and information are available within the main text and supplemental information.
• This paper does not report original code.
• Any additional information required to reanalyze the data reported in this paper is available from the lead contact upon request.

## Acknowledgments

The research data was funded by the CEDMO NPO project (Z220312000000) under the CEDMO NPO project (RRF - the Recovery and Resilience Facility). This research was funded by the Ministry of Education, Research, Development and Youth of the Slovak Republic and the Slovak Academy of Sciences as a part of the research project VEGA No. 1/0554/24. This research was supported by the Institutional support by Charles University, Program Cooperatio (IPS FSV).

## Author contributions

N.H., conceptualization, writing – original draft, writing – review & editing, supervision, visualization. B.G., formal analysis, methodology, data curation, writing – original draft, writing – review & editing, validation, visualization. V.M., methodology, writing – original draft, writing – review & editing, validation, visualization, supervision. M.K., formal analysis, software, data curation, visualization.

## Declaration of interests

The authors declare no competing interests.

## STAR★Methods

### Key resources table

**Table:** 

REAGENT or RESOURCE	SOURCE	IDENTIFIER
**Deposited data**		

Data Source – Survey responses on climate change mitigation technologies	This paper	Supplemental information

**Software and algorithms**		

Statistical Software – IBM SPSS Statistics, Version 30	IBM^25^	List of references
Regression Model – Descriptive Statistics and Binary logistic regression	Galton, F.^34^	List of references
Outcome Variable – Perceived meaningfulness of technologies (binary)	This paper	Supplemental information – outcome models
Predictor Variables – Sociodemographic factors (e.g., age, education, income)	This paper	Supplemental information – outcome models

**Other – examined techniques**		

Reforestation	Pita et al.^27^ Baum et al.^26^	List of references List of references
Afforestation	Psistaki et al.^28^ Baum et al.^26^	List of references List of references
Capture of CO_2_	Flight et al.^29^ Keith et al.^30^ Baum et al.^26^	List of references List of references List of references
Energy production from biomass	Fuss et al.^31^ Baum et al.^26^	List of references List of references
Stratospheric sulphate aerosol dispersal	Crutzen et al.^32^ Baum et al.^26^	List of references List of references
Reflective mirrors in outer space	Baum et al.^26^ Baum et al.^33^	List of references List of references

### Experimental model and study participant details

The contracting authority for data collection was the Charles University, the research workplace CEDMO NPO respectively and supplier was agency MEDIAN. The submission of the questionnaire was prepared by the contracting authority and its final version was compiled in collaboration with the supplier. The questionnaire was administered electronically. The respondents were selected from the MEDIAN online panel. The respondents were recruited according to the quota regulation approved by the contracting authority.

#### Ethics

All subjects were informed about the study, and all provided informed consent. The study procedures were carried out in accordance with the Declaration of Helsinki and was approved by ethical committee of General University Hospital Prague (IORG0002175 – General University Hospital in Prague, IRB00002705 a Federalwide Assurance FWA00029052).

### Method details

The research study was created according to the research questions included in the research questionnaire within the CEDMO Trends project (eleventh wave). The inspiration for the compilation of the research questions was the study: Baum, C.M.,^26^ Pita et al.,^27^ Psistaki et al.^28^ Flight and Tait,^29^ Keith et al.,^30^ Fuss et al.,^31^ Crutzen et al.,^32^ Baum et al.^33^

#### Fundamental parameters of the research

Name of the research: CEDMO Trends: Czech society in changing times (11th wave)

Date of main data collection: 9 February 2024 – 25 February 2024

Target group: population 16+

Method of respondents selection: quota selection

Support for setting quotas: Czech Statistical Office

Monitored quotas: sex, age, education, region, residence size, Internet use, employment status, voting behaviour in the 2021 parliamentary elections and the 2023 presidential elections

Data collection method: Computer-Assisted Web Interviewing (CAWI)

Total valid questionnaires: 3,007 (3,026 before not suitable ones)

#### Data collection

Respondents were selected from the previously constructed panel, built from the database of respondents of the Median online panel before the initiation of the first wave.

This panel of N = 4,000 was compiled according to the regulations approved by the authority. The process of adjusting the panel so that it represented the general population of the Czech Republic regarding the determined characteristics occurred after the first and second wave of the data collection and it was already completed before the start of the third wave. It follows eliminating a part of the overrepresented strongest Internet users and on the contrary, adding the underrepresented weakest Internet users. Beginning with the fourth wave, all respondents from the constructed panel are addressed. Although, a total of 98 respondents have already requested the termination of collaboration.

#### A total number of the addressed respondents was in the eleventh wave 3,902, of which:

(1) 730 respondents did not respond to the invitation to fill in the questionnaire – this means the response rate is at a level of 81.3 %;
(2) 38 respondents, who entered the questionnaire, was excluded by the filter;
(3) 119 respondents did not complete the questionnaire;
(4) 3,026 respondents completed the entire questionnaire;
(5) 19 questionnaires were deleted due to the too short completion time;
(6) 3007 questionnaires were included in the processing.

No additional adjustments were made to the questionnaire after the start of data collection. Each respondent was addressed with an offer to participate in the research via e-mail and had at least three days to fill out the questionnaire. Roughly, a third of the respondents were urged at least once. The respondent received a reward corresponding to the payment standard of the MEDIAN agency after completing the questionnaire. A share of 15 % of respondents gave up their reward in favour of a charitable organisation. The average length of completing the questionnaire was 38.3 minutes.

#### Survey instrument

Survey responses were collected from a representative sample of the general population. The study targeted individuals across various age groups, educational backgrounds, geographic regions, and occupational statuses. The survey was conducted online to ensure a broad reach and to facilitate standardized data collection.

(1) The semantic differential scale (-6 to +6) was utilized to gauge attitudes toward the environmental benefits, cost-effectiveness, long-term viability, and potential for international cooperation of different climate change mitigation technologies.
(2) The Likert scale (1 to 6) was used to assess the perceived meaningfulness of these technologies.
(3) Participants answered demographic questions regarding age, gender, education, region of residence, city size, economic activity, and frequency of internet usage.

#### Overview of analysis

Mean and median scores were calculated for each concerned technology based on survey responses.

Boxplots were generated to visualize response distributions.

Six separate binary logistic regressions were conducted to analyse how sociodemographic factors shape the perceived meaningfulness of various climate change mitigation technologies. The dependent variable in each model was the perceived meaningfulness of a given technology, dichotomized based on semantic differential scale. Ratings from 1 to 3 were classified as "meaningful" (assigned a value of 1), while ratings from 4 to 6 were classified as "not meaningful" (assigned a value of 0). This transformation allowed for logistic regression analysis, which estimates the likelihood of a respondent considering a technology meaningful.

### Quantification and statistical analysis

(1) **Data Processing**: Responses were cleaned, and missing data were handled using listwise deletion.
(2) **Variable Selection**: Sociodemographic predictors included age, gender, education, and income.
(3) **Model Specification**: For each technology, a logistic regression model was estimated where the dependent variable was dichotomized (meaningful vs. not meaningful).
(4) **Model Evaluation**: We assessed model fit using McFadden’s R^2^, and the goodness-of-fit was checked using the Hosmer-Lemeshow test.

#### Limitations

(1) Self-reported data may introduce response biases.
(2) The dichotomization of Likert-scale ratings may reduce distinction in perception differences.
(3) Results may not generalize beyond the surveyed population.

## Supplemental information

Data S1. Data on the climate change
